# Differential effects of short-term *β* agonist and growth hormone treatments on expression of myosin heavy chain IIB and associated metabolic genes in sheep muscle

**DOI:** 10.1017/S175173111400233X

**Published:** 2014-09-12

**Authors:** K. M. Hemmings, Z. C. T. R. Daniel, P. J. Buttery, T. Parr, J. M. Brameld

**Affiliations:** School of Biosciences, Division of Nutritional Sciences, The University of Nottingham, Sutton Bonington Campus, Leicestershire, LE12 5RD, UK

**Keywords:** *β* agonist, growth hormone, muscle fibre type, myosin heavy chain, sheep

## Abstract

Growth hormone (GH) and *β* agonists increase muscle mass, but the mechanisms for this response are unclear and the magnitude of response is thought to vary with age of animal. To investigate the mechanisms driving the muscle response to these agents, we examined the effects of short-term (6 day) administration of GH or cimaterol (a *β*2-adrenergic agonist, BA) on skeletal muscle phenotype in both young (day 60) and mature (day 120) lambs. Expression of myosin heavy chain (MyHC) isoforms were measured in *Longissimus dorsi* (LD), *Semitendinosus* (ST) and *Supraspinatus* (SS) muscles as markers of fibre type and metabolic enzyme activities were measured in LD. To investigate potential mechanisms regulating the changes in fibre type/metabolism, expression or activity of a number of signalling molecules were examined in LD. There were no effects of GH administration on MyHC isoform expression at either the mRNA or protein level in any of the muscles. However, BA treatment induced a proportional change in MyHC mRNA expression at both ages, with the %MyHCI and/or IIA mRNA being significantly decreased in all three muscles and %MyHCIIX/IIB mRNA significantly increased in the LD and ST. BA treatment induced *de novo* expression of MyHCIIB mRNA in LD, the fastest isoform not normally expressed in sheep LD, as well as increasing expression in the other two muscles. In the LD, the increased expression of the fastest MyHC isoforms (IIX and IIB) was associated with a decrease in isocitrate dehydrogenase activity, but no change in lactate dehydrogenase activity, indicating a reduced capacity for oxidative metabolism. In both young and mature lambs, changes in expression of metabolic regulatory factors were observed that might induce these changes in muscle metabolism/fibre type. In particular, BA treatment decreased PPAR-*γ* coactivator-1*β* mRNA and increased receptor-interacting protein 140 mRNA. The results suggest that the two agents work via different mechanisms or over different timescales, with only BA inducing changes in muscle mass and transitions to a faster, less oxidative fibre type after a 6-day treatment.

## Implications

This study provides mechanistic information on the anabolic responses to growth hormone and *β*-adrenergic agonist in sheep muscle. It therefore has implications for the use of these agents in farm animals, which is licensed in some parts of the world (e.g. United States, Australia) but not others (e.g. EU). Regarding cross-species comparisons, there are clear differences in responses between sheep and rodents, with sheep appearing to respond in a manner more similar to humans than rats. Indeed, the fibre type composition of sheep muscle is more similar to humans than rats, suggesting that sheep may be a better model organism for conducting skeletal muscle studies.

## Introduction

Administration of growth hormone (GH) or *β*-adrenergic agonist (BA) results in the repartitioning of nutrients, increasing skeletal muscle mass and decreasing fat mass (Buttery and Dawson, 1990). The effectiveness of these agents is thought to be reduced in young, rapidly growing animals compared with those approaching mature BW (Bell *et al*., 1998). In addition, the increase in skeletal muscle mass may be dependent upon muscle fibre type composition or involve fibre type transitions (Lefaucheur and Gerrard, 2000). In recent years, measurement of myosin heavy chain (MyHC) isoforms has been used to indicate the fibre type composition of skeletal muscles (Sazili *et al*., 2005). In sheep and cattle, the predominant MyHC isoforms expressed in adult animals are types I, IIA and IIX, with the ‘fastest, most glycolytic’ isoform, MyHCIIB, being rarely detected in these species (Hemmings *et al*., 2009; Picard and Cassar-Malek, 2009).

The factors involved in regulating fibre type transitions are poorly understood, particularly those relating to fast glycolytic fibre types. Rodent studies have identified a number of potential regulatory signalling molecules, including the myogenic regulatory factors (MRFs; Hughes *et al*., 1993) and the signalling enzymes, calcineurin (CaN) and calmodulin kinase II (CaMKII; Chin, 2005; Bassel-Duby and Olson, 2006). The Six1/Eya1 protein complex has been shown to be important in the regulation of the fast glycolytic fibre type in rodents (Grifone *et al*., 2004), but their role in other species is not known. In addition, the peroxisome proliferator-activated receptor (PPAR) transcription factors, along with the PPAR-*γ* coactivator-1 (PGC-1) family of transcriptional coactivators and the transcriptional co-repressor, receptor-interacting protein 140 (RIP140), are all known to influence muscle metabolic gene expression in rodents (Lin, 2009), but again their role in other species is not known.

The objective of the current study was therefore to compare the effects of short-term (6 day) treatment with GH or BA on enzyme activities and expression of genes associated with skeletal muscle metabolism and fibre type in young (rapidly growing) and mature (fattening) lambs. BA had previously been shown to alter MyHC isoform expression after 1 week of treatment in pigs (Gunawan *et al*., 2007) and therefore this short-term (6 day) treatment was chosen in order to investigate the initial (inductive) response to these agents and potential regulatory mechanisms involved.

## Material and methods

Sheep studies were approved by the University of Nottingham Local Ethical Review Committee and were carried out in accordance with the UK Home Office guidelines (Animals (Scientific Procedures) Act, 1986).

### Sample collection and preparation

Male (Mule×Charolais) twin lambs were group housed with their mothers until 53±4 days of age, when they were weaned and pairs were split into day 60 (D60) and day 120 (D120) groups. These ages were chosen based on a previous (unpublished) study at the University of Nottingham, showing that D60 was within the rapid growth phase and D120 represented mature lambs with reduced growth rates and increased backfat deposition. Creep feed (48.48% barley, 27% soya, 15% nutritionally improved straw, 5% molassed feed meal, 2.5% vitamins and minerals, 1% limestone, 0.5% salt, 0.5% vegetable oil and 0.02% Minsal g109 E50) was introduced ∼28 days before weaning and lambs were given free access from weaning onwards. Lambs were individually penned with *ad-libitum* access to creep feed for 7 days before the commencement of treatments. The D60 and D120 groups were split into three treatment groups, ensuring that the mean initial BWs for each group were not significantly different. The control group (CO, *n*=11) were fed the creep diet *ad libitum*; the GH group (*n*=10) were fed the same diet *ad libitum* and administered a prolonged release bovine GH formulation (POSILAC, Monsanto, St Louis, Missouri, USA) on the 1^st^ day of treatment by a single subcutaneous injection ∼5 cm from the nape of the neck, at a dose of 3.75 mg/kg BW, as described by McLaughlin *et al*. (1994). The BA group (*n*=10) were fed the same creep diet *ad libitum*, but containing the *β*2-adrenergic agonist, cimaterol, at 10 ppm (10 mg/kg). The treatments were for 6 days, starting at 60±4 or 120±4 days of age. This short-treatment period was chosen in order to investigate the initial (inductive) response to these agents, particularly in terms of intracellular signalling. At slaughter, blood was collected and plasma prepared, while samples of the *Longissimus dorsi* (LD, from the thoracic region at the 10th rib), *Supraspinatus* (SS) and *Semitendinosus* (ST) muscles from the left side of the carcass, were snap frozen in liquid nitrogen and stored at −80°C; these were subsequently crushed and mixed before further analyses. LD and ST muscles were chosen for their commercial importance and as representative ‘fast’ muscles, whereas SS was selected as a ‘slower’ muscle type (Hemmings *et al*., 2009). The whole LD, ST, SS, *Vastus lateralis* (Vlat) and *Vastus intermedius* (Vint) muscles were dissected from the right side of the carcass and weighed along with the liver, all within 1 h of slaughter.

### Plasma IGF-I and glycerol concentrations and LD skeletal muscle glycogen content

Plasma IGF-I concentrations were determined using an enzyme-linked immunosorbent assay kit (Diagnostic Systems Laboratories, Oxford, UK) and plasma glycerol concentrations were measured using the Serum Triglyceride determination kit (Sigma Aldrich, Poole, UK), both procedures were carried out according to the manufacturers’ instructions. Acid-soluble muscle glycogen was determined using a protocol adapted from Dreiling *et al*. (1987). Briefly, 1 g of muscle was homogenised in 4 ml of 8% (vol/vol) perchloric acid (PCA), then 1 ml was removed and centrifuged at 14 000×**g** at 4°C for 10 min. To neutralise the supernatant, 133 µl was mixed with 150 µl of saturated sodium bicarbonate (5 M) solution and 167 µl of 200 mM sodium acetate buffer, pH 4.8. A range of glycogen standards were prepared in neutralised PCA containing 0 to 300 µg of bovine liver glycogen (Sigma Aldrich) in a 200 µl volume. Amyloglucosidase (5 µl of 80 U/ml, Sigma Aldrich) was added to 200 µl of each standard or neutralised sample supernatant and incubated at 37°C for 30 min to release the glucose from the glycogen. Samples were heated at 100°C for 5 min to stop the reaction and then centrifuged at 14 000×**g** for 10 min at room temperature. Glucose concentrations were determined using Enzymatic Glucose Reagent (Thermo Electron Corporation, Fife, UK), by measuring the absorbance at 550 nm on a 680 XR 96-well microplate reader (Bio-Rad, Hemel Hempstead, UK).

### Skeletal muscle total RNA extraction and cDNA synthesis

Total RNA was extracted from LD, SS and ST muscle samples using TRIZOL (Invitrogen, Paisley, UK), according to the manufacturer’s instructions, and then treated with DNase (Promega, Southampton, UK). Following DNase treatment, RNA was quantified using the Nanodrop spectrophotometer ND-1000 (Thermo Scientific, Wilmington, DE, USA) and RNA integrity was checked by running samples on a 1% agarose gel and visualising the 28S and 18S ribosomal RNA bands. First strand complementary DNA (cDNA) was synthesised from 1 µg of total RNA using random hexamer primers and Moloney murine leukemia virus reverse transcriptase (Promega) in a final volume of 25 μl (Hemmings *et al*., 2009).

### MyHC isoform expression

The relative expression of the adult MyHC isoforms (MyHCI, IIA, IIX and IIB) were determined at both the mRNA and protein level, as described previously (Hemmings *et al*., 2009). Briefly, SDS–PAGE separation of MyHC isoforms was carried out to determine percentage MyHC protein expression, comparing samples to a bovine positive control (*Longissimus thoracis*) sample (kindly provided by Dr Brigitte Picard, INRA, France) previously shown to express all four adult MyHC isoforms (Picard and Cassar-Malek, 2009). Quantitative real-time PCR was carried out on muscle cDNA to determine percentage MyHC mRNA expression, using ovine specific primers and probes for MyHCI, IIA and IIX (Supplementary Table S1). The relative percentage of each isoform was calculated as described previously (Hemmings *et al*., 2009). Primers and probes designed to the available sequence for the ovine MyHCIIX isoform would be expected to generate amplicons from any MyHCIIB transcripts present as well, as this region is identical in the porcine sequences for MyHCIIX and IIB, therefore expression is stated as MyHCIIX/IIB.

The mRNA expression of the MyHCIIB isoform was analysed by semi-quantitative RT-PCR using published primers previously described as detecting only ovine MyHCIIB (Vuocolo *et al*., 2007). MyHCIIB expression was compared with the semi-quantitative RT-PCR analysis of MyHCIIX+IIB combined (using the primers in Supplementary Table S1).

### Metabolic enzyme activities

Isocitrate dehydrogenase (ICDH) and lactate dehydrogenase (LDH) activities were determined in LD homogenates using the methods described by Brandstetter *et al*. (1998) as an indication of oxidative and glycolytic metabolism, respectively.

### CaN and calmodulin kinase activities

Protein content was determined in LD homogenates using the Bradford assay (Bradford, 1976) and samples adjusted to constant protein before assay. CaN activity was determined in skeletal muscle homogenates using the BIOMOL CaN cellular assay kit plus (Enzo Life Sciences Ltd, Exeter, UK). CaMKII activity was determined using the SignaTECT calcium/calmodulin-dependent protein kinase (CaMKII) Assay kit (Promega).

### Real-time PCR analysis of signalling molecule gene expression

Ovine specific primers and probes were designed using Primer Express software (Applied Biosystems, Warrington, UK) and are shown in Supplementary Table S1. LD cDNA was diluted 1 in 4 and a pool of samples generated and serially diluted to generate a standard curve. Each sample cDNA was further diluted 1 in 8 and 5 μl was used for real-time PCR reactions (in triplicate), using 384 well plates and 1X Lightcycler 480 probes master PCR mastermix (Roche, Burgess Hill, UK) in 15 μl final reaction volume (containing 0.3 μM of each primer and 0.2 μM of probe). Reactions were run on the Lightcycler 480 PCR machine (Roche) with the following conditions: 95°C for 10 min and then 45 cycles of 95°C for 10 s, 60°C for 50 s and 72°C for 1 s, during which fluorescence was measured. Crossing point (Cp) values were determined using the second derivative maximum method and quantified relative to the standard curve (Brown *et al*., 2012). Supplementary Table S1 includes the primers used for PCR. After being unable to find a suitable housekeeping gene for normalising the data, it was decided to express the real-time data relative to the equivalent quantity of RNA in the reaction, given that the total RNA concentrations had been normalised before cDNA synthesis.

### Statistical analysis

Data are presented as means or as a percentage value. All data had a normal distribution. Data for each age group was analysed separately by one-way ANOVA (Genstat release 11.1. (Lawes Agricultural Trust, Hertfordshire, UK)), blocking for gel where appropriate. s.e.d. values are included for the ANOVA. When significant differences were observed (with ANOVA), a *post hoc* Dunnett’s test was then used to compare each treatment group to the control. If not significant (i.e. *P*>0.05), the *P*-values shown relate to the ANOVA, but in the text significant differences are indicated using *P*-values for the Dunnett’s test. Tables include the *P*-values for the ANOVA, with * used to indicate significant differences for the Dunnett’s test.

## Results

### Lamb responsiveness to BA and GH treatments

The effects of a 6-day treatment with BA or GH on lamb carcass characteristics are shown in Table 1. Neither treatment significantly altered BWs (*P*>0.05) and there were no significant differences in any muscle weights at D60 or in LD and VInt muscle weights at D120 (*P*>0.05). However, BA treatment resulted in significant increases in SS and ST muscle weights at D120 (*P*<0.05), with VLat weights also tending to be higher in the BA treated older lambs (*P*=0.058). As expected, GH significantly increased liver weights (*P*<0.05; Table 1) and plasma IGF-1 levels (*P*<0.05; Table 2) at both ages; while BA significantly reduced LD glycogen content at both ages (*P*<0.05; Table 2). Surprisingly, plasma glycerol levels were unaffected (*P*>0.05; Table 2) by treatment at both ages.

**Table 1:** The effects of short-term (6 day) BA or GH treatment of lambs at 60 or 120 days of age on lamb carcass characteristics

	D60					D120				
Trait	CO	BA	GH	s.e.d.	*P*-value	CO	BA	GH	s.e.d.	*P*-value
*n*	11	10	10			11	10	10		
BW (kg)	23.9	23.9	24.5	1.4	0.863	43.0	46.4	44.3	1.6	0.122
Liver (g)	475	460	567^a^	32	0.005	928	926	1101^a^	55	0.004
Muscle weights (g)										
* Longissimus dorsi*	305	306	327	27	0.655	592	667	632	35	0.108
* Supraspinatus*	53.5	55.2	55.9	3.9	0.812	98.5	116.4^a^	98.0	6.3	0.009
* Semitendinosus*	63.3	67.1	65.0	4.3	0.667	112.4	131.2^a^	120.4	6.6	0.023
* Vastus lateralis*	85.4	88.0	89.3	6.3	0.815	164.5	180.3	163.8	7.5	0.058
* Vastus intermedius*	28.5	27.5	30.9	2.3	0.331	51.7	55.7	52.4	3.6	0.487

BA=*β* agonist; GH=growth hormone; CO=control.

a Significantly different to control (Dunnett’s test; *P*<0.05).

**Table 2:** The effects of short-term (6 day) BA or GH treatment of lambs at 60 or 120 days of age on plasma IGF-1 and glycerol concentrations and muscle glycogen content

	D60					D120				
Trait	CO	BA	GH	s.e.d.	*P*-value	CO	BA	GH	s.e.d.	*P*-value
*n*	11	10	10			11	10	10		
IGF-1 (ng/ml)	375	373	928^a^	129	<0.001	972	964	2092^a^	127	<0.001
Glycerol, (μg/ml)	6.3	8.2	7.3	1.1	0.191	8.2	9.4	9.5	2.4	0.826
LD Glycogen (mg/g)	25.5	19.0^a^	23.8	1.6	<0.001	21.6	18.3^a^	24.2	1.3	<0.001

BA=*β* agonist; GH=growth hormone; CO=control; LD=*Longissimus dorsi*.

a Significantly different to control (Dunnett’s test; *P*<0.05).

### Effects of BA and GH on MyHC expression in LD, SS and ST muscles

As an indication of the effects of treatment on the fibre type composition of the LD, SS and ST muscles, MyHC expression was determined at both the mRNA and protein levels. GH treatment had no effects (*P*>0.05) on MyHC isoform mRNA (Table 3) or protein (Table 4) expression in any of the muscles studied. In contrast, there were significant effects of BA treatment on percentage MyHC isoform mRNA expression in all three muscles at both ages (Table 3). At D60, BA treatment decreased the %MyHCI mRNA in the LD (*P*<0.05), but there were no effects in the SS and ST muscles (*P*>0.05) and there were no effects on %MyHCI mRNA expression in any muscle at D120 (*P*>0.05). BA treatment consistently reduced %MyHCIIA mRNA expression in all three muscles at both ages (*P*<0.05). Interestingly, BA treatment increased the %MyHCIIX/IIB mRNA expression in the two fast muscles, LD and ST, at both ages (*P*<0.05). The response observed in the SS muscle was similar, but either not significant at D120 or appeared to only increase relative to the GH group at D60.

**Table 3:** The effects of short-term (6 day) BA or GH treatment of lambs at 60 or 120 days of age on MyHC isoform mRNA expression in different muscles

		D60					D120				
Muscle	Isoform (%)	CO	BA	GH	s.e.d.	*P*-value	CO	BA	GH	s.e.d.	*P*-value
	*n*	10 to 11	10	10			10 to 11	10	9 to 10		
*Longissimus dorsi*	MyHCI	7.2	3.9^a^	6.6	0.8	<0.001	6.8	5.0	8.1	1.9	0.277
	MyHCIIA	26.9	7.0^a^	34.1	3.4	<0.001	21.1	5.2^a^	25.4	3.6	<0.001
	MyHCIIX/IIB	65.9	89.1^a^	59.3	3.6	<0.001	72.1	89.8^a^	66.5	4.2	<0.001
*Supraspinatus*	MyHCI	34.7	37.7	42.3	8.8	0.693	56.6	51.6	45.5	9.6	0.520
	MyHCIIA	21.2	9.1^a^	27.9	3.8	<0.001	15.8	5.3^a^	17.2	2.1	<0.001
	MyHCIIX/IIB	44.1	53.2	29.8	7.3	0.012	27.6	43.1	37.3	9.1	0.243
*Semitendinosus*	MyHCI	13.5	8.9	11.7	2.4	0.161	11.2	14.6	13.5	2.2	0.259
	MyHCIIA	20.6	4.3^a^	25.5	2.8	<0.001	17.1	2.7^a^	20.4	2.6	<0.001
	MyHCIIX/IIB	66.0	86.8^a^	62.7	4.0	<0.001	71.7	82.7^a^	66.2	3.8	<0.001

BA=*β* agonist; GH=growth hormone; MyHC=myosin heavy chain; CO=control.

a Significantly different to control (Dunnett’s test; *P*<0.05).

**Table 4:** The effects of short-term (6 day) BA or GH treatment of lambs at 60 or 120 days of age on MyHC isoform protein expression in different muscles

		D60					D120				
Muscle	Isoform (%)	CO	BA	GH	s.e.d.	*P*-value	CO	BA	GH	s.e.d.	*P*-value
	*n*	11	10	10			11	10	10		
*Longissimus dorsi*	MyHCI	10.2	9.4	9.4	1.1	0.680	8.1	8.9	8.2	1.0	0.802
	MyHCIIA	33.3	29.9	35.0	1.8	0.028	31.9	30.7	33.3	1.5	0.236
	MyHCIIX/IIB	56.5	60.7	55.6	2.3	0.060	60.0	60.4	58.4	1.8	0.533
*Supraspinatus*	MyHCI	42.1	45.4	42.0	8.2	0.852	48.4	34.2	47.2	8.5	0.166
	MyHCIIA	20.6	19.2	22.0	2.0	0.387	20.3	18.2	17.7	1.6	0.429
	MyHCIIX/IIB	37.3	35.3	36.0	6.7	0.916	31.3	47.6	35.1	7.6	0.086
*Semitendinosus*	MyHCI	11.0	9.0	8.7	1.8	0.308	7.3	9.2	6.9	1.7	0.347
	MyHCIIA	26.3	26.2	29.1	1.5	0.142	25.0	24.0	24.8	2.0	0.920
	MyHCIIX/IIB	62.7	64.8	62.2	2.8	0.578	67.8	66.8	68.2	3.3	0.870

BA=*β* agonist; GH=growth hormone; MyHC=myosin heavy chain; CO=control.

The effects of BA treatment on MyHC isoform mRNA expression were not observed at the protein level (Table 4). However, BA treatment did appear to reduce %MyHCIIA protein in the LD at D60, but only relative to the GH group, and BA tended to increase %MyHCIIX protein in the LD at D60 and SS at D120 (Table 4).

**Figure 1:**
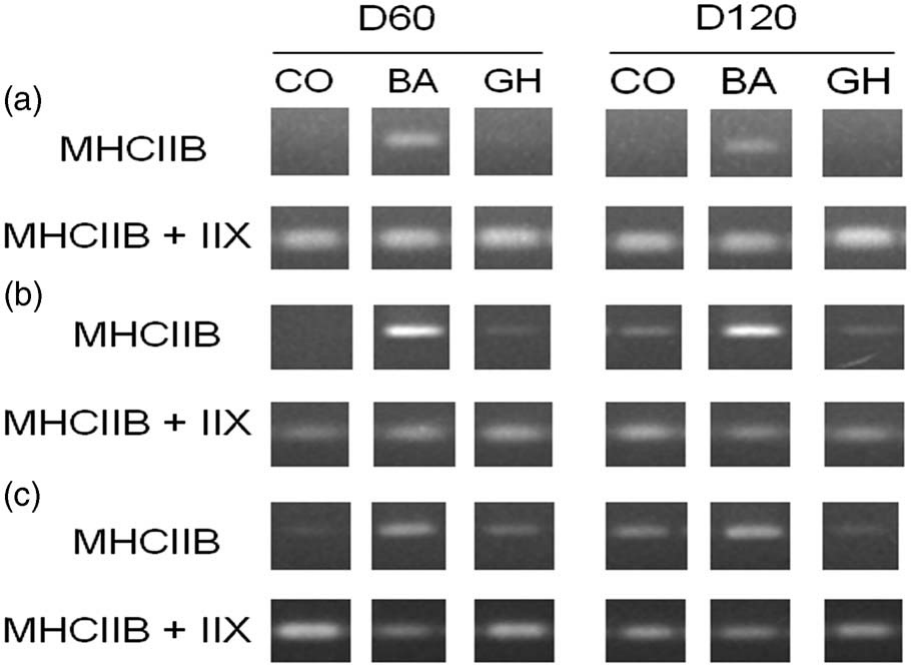
Representative semi-quantitative RT-PCR for myosin heavy chain (MyHC) IIB and IIB+IIX mRNAs in (a) *Longissimus dorsi* (LD), (b) *Supraspinatus* (SS) and (c) *Semitendinosus* (ST) muscles from D60 and D120 lambs. The 6-day administration of *β* agonist (BA) induced the expression of MyHCIIB mRNA in the LD, with no expression of this transcript in either the control (CO) or Growth Hormone (GH) treated lambs. BA also increased MyHCIIB expression in the SS and ST muscles.

**Figure 2:**
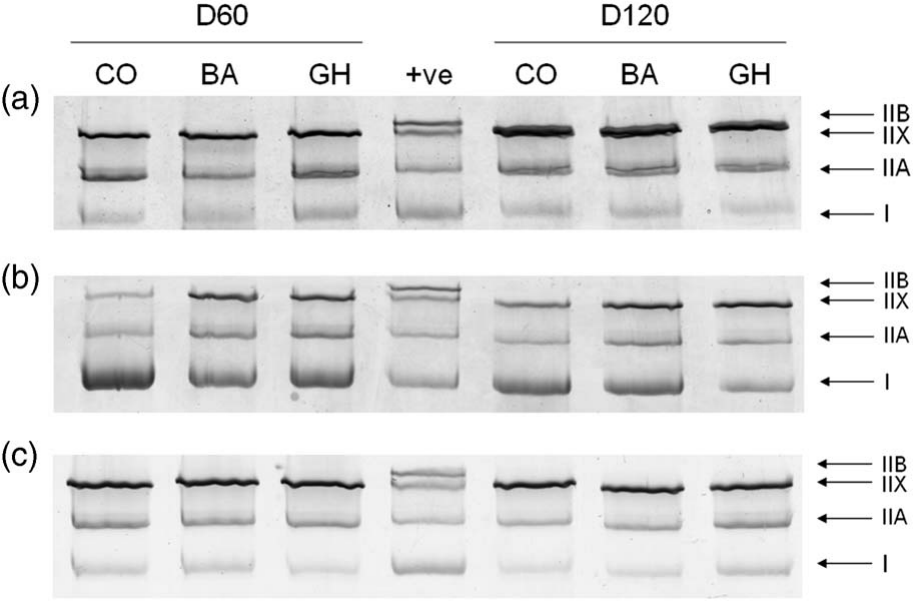
Representative SDS–PAGE separation of myosin heavy chain (MyHC) isoforms in (a) *Longissimus dorsi* (LD), (b) *Supraspinatus* (SS) and (c) *Semitendinosus* (ST) muscles from D60 and D120 lambs. +ve represents a bovine *Longissimus thoracis* sample containing all four adult MyHC isoforms (kindly provided by Dr Brigitte Picard, INRA, France). The arrows indicate the position of MyHC isoforms as determined from this bovine +ve control sample.

As BA treatment significantly increased %MyHCIIB/IIX mRNA in the LD and ST muscles, expression of MyHCIIB mRNA was determined by semi-quantitative RT-PCR and compared with the expression of MyHCIIX and IIB mRNA combined (Figure 1). MyHCIIB mRNA was induced by BA treatment in the LD of lambs at both ages, and expression of the MyHCIIB isoform was increased by BA in the SS and ST muscles. However, MyHCIIB was not detected at the protein level in any of the muscles studied (Figure 2).

Overall, the changes in MyHC isoform expression indicate that muscles from BA-treated lambs were in transition towards a faster, more glycolytic fibre type composition, with MyHCIIA fibres switching to MyHCIIX and/or IIB fibres. Because of the observed induction of *de novo* mRNA expression of the MyHCIIB isoform in the LD in response to BA treatment, further characterisation of the metabolic response and postulated intracellular signalling mechanisms were conducted in that muscle.

### Effects of BA and GH on metabolic enzyme activities of LD

ICDH and LDH activities were determined in the LD as an indication of oxidative and glycolytic metabolic activities, respectively (Table 5). ICDH activity was significantly decreased in BA treated lambs at both ages (*P*<0.05); but there were no effects of treatment on LDH activities (*P*>0.05, Table 5). Overall, the LD appears to be changing to a less oxidative metabolism, which would agree with the changes in MyHC isoform mRNA expression described previously.

**Table 5:** The effects of short-term (6 day) BA or GH treatment of lambs at 60 or 120 days of age on metabolic and signalling enzyme activities in the Longissimus dorsi

	D60					D120				
Trait	CO	BA	GH	s.e.d.	*P*-value	CO	BA	GH	s.e.d.	*P*-value
*n*	10 to 11	7 to 9	10			10 to 11	9 to 10	10		
ICDH activity (mOD/min per mg tissue)	49.3	33.9^a^	49.4	4.9	0.004	37.3	28.2^a^	33.1	3.4	0.041
LDH activity (mOD/min per mg tissue)	510	489	519	86	0.928	637	618	652	114	0.957
CaMKII (pmol/min per µg protein)	11.5	14.3	10.9	2.1	0.246	13.8	14.9	11.5	1.8	0.160
CaN (×10^−3^) arbitrary units	49.5	43.0	44.4	15.5	0.901	34.1	27.1	22.5	11.1	0.562

BA=*β* agonist; GH=growth hormone; CO=control; ICDH=isocitrate dehydrogenase; LDH=lactate dehydrogenase; CaMKII=calmodulin kinase II; CaN=calcineurin.

a Significantly different to control (Dunnett’s test; *P*<0.05).

### Effects of BA and GH on signalling molecules and regulatory factors in LD

The enzyme activity (Table 5) or mRNA expression (Table 6) of selected mediators thought to influence muscle contractile and/or metabolic properties were determined in LD. Surprisingly, the activities of the calcium dependent signalling enzymes, CaN and CaMKII, were unaffected by treatment at either age (*P*>0.05 for all, Table 5). There were no significant effects of treatment on PGC-1*α* mRNA expression at either age (*P*>0.05, Table 6), whereas PGC-1*β* mRNA expression was significantly reduced by both BA and GH treatment at D60 (*P*<0.05, Table 6), but only by BA treatment at D120 (*P*<0.05, Table 6). In contrast, RIP140 mRNA expression was significantly increased by BA treatment at both ages (*P*<0.05, Table 6). GH treatment reduced PPAR*α* mRNA expression in the D120 lambs (*P*<0.05, Table 6), but there were no effects at D60 (*P*>0.05, Table 6). Similarly, BA increased PPAR*δ* mRNA expression in the D120 lambs (*P*<0.05, Table 6), but there were no effects in the D60 lambs (*P*>0.05, Table 6). PPAR*γ* mRNA expression was unaffected by treatment (*P*>0.05 for all, Table 6) at either age. Surprisingly, Six1 and Eya1 mRNA expression were significantly decreased by BA treatment in both age groups (*P*<0.05, Table 6). There were no significant treatment effects (*P*>0.05, Table 6) on Myf-5 or MRF4 mRNA expression at either age, nor on MyoD mRNA expression in the D60 lambs, but BA treatment reduced MyoD mRNA expression in the D120 lambs (*P*<0.05, Table 6). In contrast, BA treatment increased myogenin mRNA expression at both ages (*P*<0.05), suggesting a possible effect on myogenic/satellite cell differentiation.

**Table 6:** The effects of short-term (6 day) BA or GH treatment of lambs at 60 or 120 days of age, on mRNA expression of regulators of muscle contractile and metabolic properties in the Longissimus dorsi

	D60					D120				
Gene mRNA target^a^	CO	BA	GH	s.e.d.	*P*-value	CO	BA	GH	s.e.d.	*P*-value
*n*	11	10	10			11	10	10		
PPAR*α*	4.46	3.82	3.94	0.31	0.096	4.53	3.74	2.91^b^	0.35	<0.001
PPAR*δ*	4.30	5.75	4.08	0.86	0.124	3.82	5.14^b^	4.22	0.35	0.002
PPAR*γ*	4.85	2.91	4.39	1.76	0.516	4.46	3.45	4.14	0.48	0.111
PGC1*α*	4.53	4.36	3.70	0.77	0.521	4.28	4.67	3.85	0.58	0.376
PGC1*β*	5.11	2.87^b^	3.85^b^	0.45	<0.001	4.16	2.68^b^	3.42	0.44	0.007
RIP140	3.59	5.58^b^	4.30	0.66	0.015	3.86	5.63^b^	3.98	0.55	0.005
Six1	4.31	2.58^b^	4.11	0.41	<0.001	4.66	2.24^b^	4.24	0.27	<0.001
Eya1	4.38	2.73^b^	4.29	0.53	0.006	5.35	2.93^b^	5.04	0.69	0.002
MyoD	2.95	2.66	2.73	0.40	0.735	3.00	2.01^b^	3.28	0.39	0.008
Myogenin	3.34	4.65^b^	3.65	047	0.023	3.15	4.54^b^	3.23	0.40	0.002
MRF4	3.02	4.47	3.59	1.36	0.552	4.08	4.14	5.06	0.63	0.230
Myf-5	2.71	3.69	3.22	0.40	0.059	4.33	3.71	3.71	0.57	0.436

BA=*β* agonist; GH=growth hormone; CO=control; PPAR=peroxisome proliferator-activated receptor; PGC=PPAR-*γ* coactivator; RIP=receptor-interacting protein; MRF=myogenic regulatory factor.

a Values for gene targets expressed as arbitrary units per unit total RNA.

b Significantly different to control (Dunnett’s test; *P*<0.05).

## Discussion

The major findings from this sheep study relate to BA administration which, at both ages, induced changes that are indicative of muscle fibre type transitions towards a fast glycolytic phenotype, with *de novo* expression of MyHCIIB mRNA being observed. These changes in MyHC isoform mRNA expression corresponded with a decrease in oxidative metabolism, which are likely induced by the increase in RIP140 and corresponding decrease in PGC-1*β* mRNA expression.

### Responsiveness to agents

Both GH and BA have previously been shown to induce skeletal muscle hypertrophy and have the effect of repartitioning nutrients from adipose tissue towards skeletal muscle; but they are thought to do this via different mechanisms (National Research Council, 1994). The majority of anabolic effects exerted by GH are thought to be mediated indirectly via the stimulation of IGF-1 production by the liver and other tissues (Bell *et al*., 1998), whereas the effects of GH on adipose tissue are thought to be direct. In contrast, BAs are thought to act directly on tissues (particularly muscle) by binding to *β*2-adrenergic receptors (*β*2-ARs) expressed by those tissues (National Research Council, 1994). In the present study, very different effects of GH and BA treatment were observed, particularly on factors indicative of fibre type transitions and potential regulatory factors.

Despite only a short-term (6 day) treatment, the lambs responded to both agents at both ages, although the magnitude of the responses did vary with age. At both ages, plasma IGF-1 levels and liver weights were increased by GH, which are commonly observed responses to GH administration (Brameld *et al*., 1996). As expected, BA decreased LD glycogen content at both ages. Surprisingly, neither agent altered plasma glycerol concentrations, which is commonly used as an indicator of adipose tissue lipolysis. Previous studies have shown that free fatty acid levels remain elevated during chronic BA administration, but plasma glycerol concentrations return to a basal level (O’Connor *et al*., 1991).

At D60, there were no effects of either treatment on muscle weights, whereas at D120, BA treatment significantly increased SS and ST weights. Therefore, there was an age differential in terms of muscle weight responses to BA administration, with older animals responding more. The reason for the lack of an effect on muscle weights in the younger lambs is unclear, but may be due to lower receptor numbers, a decreased binding affinity for the agonist or a more rapid desensitisation to the BA (National Research Council, 1994). It has previously been suggested that fast muscle fibres are more responsive to BA administration and that selective hypertrophy occurs in these fibres (Kim *et al*., 1987). However, there was a greater muscle weight response in the slower SS compared with the LD, which has the higher proportion of fast fibres. Of interest is the fact that a significant increase in muscle weight was observed in the ST but not the LD, which have very similar MyHC compositions. This suggests that MyHC composition may not be a major factor in determining muscle responses to BA administration. Further histochemical analysis (data not included) showed that the increased ST weights were associated with increased muscle fibre diameters (i.e. hypertrophy), with the fast fibres being mainly affected. However, we did not determine whether similar increases in fibre diameter were present in the LD as well.

### Changes in MyHC isoforms

There were no effects of GH treatment for 6 days on the relative expression of the MyHC isoforms at either the mRNA or protein levels. This may simply reflect the short (6 day) administration period, as increases in MyHCIIX protein have been reported in humans after a more chronic (12 week) period of GH treatment (Lange *et al*., 2002). In contrast, MyHC isoform transitions were observed at the mRNA level in response to BA treatment. BA induced a significant decrease in the %MyHCI and/or IIA mRNA in all three muscles studied, as well as an increase in the %MyHCIIX/IIB mRNA in the LD and ST. Importantly, BA induced *de novo* MyHCIIB mRNA expression in the LD (where it is not normally expressed) and increased MyHCIIB expression in the SS and ST. These differences were not observed at the protein level, but this may be due to the MyHC proteins having a slower rate of turnover compared with the corresponding mRNA, as the half-life of the MyHC proteins is up to 30 days (Kay, 1978). Therefore, it appears that BA is initiating changes in gene expression that are indicative of fibre type transitions, but the 6-day treatment may be insufficient to see a significant response at the protein level. Initially it was thought that MyHCIIB was not expressed in large mammals (Hämäläinen and Pette., 1995); however, it is expressed at both the mRNA and protein level in pigs (Lefaucheur *et al*., 2004). More recently MyHCIIB protein expression has been observed in muscles of the Blonde d’Aquitaine breed of bull (Picard and Cassar-Malek, 2009), but not other breeds of cattle. As in our previous paper (Hemmings *et al.*, 2009), MyHCIIB was not expressed at the protein level in any of the three sheep muscles studied. It does, however, appear to have a low level of expression at the mRNA level in the SS and ST muscles, which was subsequently increased by BA treatment.

Interestingly, our data is similar to that of Vuocolo *et al.* (2007), who showed that MyHCIIB is expressed at the mRNA level in muscles from *callipyge* sheep. Like BA treatment, the *callipyge* phenotype results in muscle hypertrophy and a switch in fibre type, with a decrease in type I fibres and a shift towards type IIX and IIB fibres. When compared with normal (wild type) sheep, the authors reported 13- and 6-fold increases in MyHCIIB expression at birth and 12 weeks of age in the *callipyge* sheep. In that study (Vuocolo *et al*., 2007), four adult MyHC isoforms were detected at the mRNA level in sheep LD skeletal muscle, with MyHCIIX being the predominant isoform and MyHCIIB being expressed at extremely low levels (⩽1% of total adult MyHC expression). The effects of BA treatment are therefore very similar to the *callipyge* sheep phenotype. Longer term administration of BA might therefore result in the *de novo* expression of MyHCIIB protein, the isoform not normally expressed at the protein level in sheep muscles (Hemmings *et al*., 2009). The observed decrease in oxidative capacity (ICDH activities) suggests that BA treatment is driving muscle fibres towards a faster, less oxidative fibre type, consistent with previous observations in rats (Polla *et al*., 2001), cattle (Cassar-Malek *et al*., 1998) and pigs (Gunawan *et al*., 2007).

It has been suggested that the switch in fibre type contributes to the muscle hypertrophy associated with BA administration, as glycolytic fibres have a larger cross-sectional area (Lefaucheur and Gerrard, 2000). An alternative theory is that the switch in fibre type might be secondary to the hypertrophy that occurs as a result of BA treatment (Gunawan *et al*., 2007). On the basis of the current study, it seems unlikely that the changes in MyHC are secondary to the hypertrophy, as changes in mRNA expression have already been observed in the LD at both ages, before significant changes in muscle weight. Our alternative hypothesis is that the fibre type transitions are simply a mechanism for the muscle to adapt to changes in availability of energy substrates, with changes in metabolism preceding changes in contractile properties. BA treatment not only stimulates glycogenolysis in skeletal muscles (Sensky *et al*., 2006), it also increases plasma glucose concentrations (Eisemann *et al*., 1988). Therefore, the muscle fibres might simply be adapting to the increased glucose availability, which may have a particularly pronounced effect in ruminants, given that they are more dependent on acetate and other volatile fatty acids for energy metabolism. The significant effects of BA on MyHC expression in the LD muscle, with a strong induction of MyHC IIB expression, provide a good model for characterising the signalling events involved in fibre type switching towards a faster phenotype.

### Effects of treatment on signalling molecules and regulatory factors

#### CaN and CaMKII activity

CaN and CaMKII are calcium dependent signalling molecules implicated in both muscle hypertrophy (Dunn *et al*., 1999; Chin, 2005) and muscle fibre type transitions (Chin, 2005; Bassel-Duby and Olson, 2006), as they are thought to regulate the slow oxidative phenotype (Naya *et al*., 2000; Chin, 2005). Both are activated by calcium, being dependent on the frequency of the intracellular fluctuations in calcium concentration (Chin *et al*., 1998; Chin, 2005). CaN has been shown to increase MyHCIIA expression (Allen and Leinwand, 2010) and potentially decrease MyHCIIX and IIB expression (Da Costa *et al*., 2007) in mouse C2C12 muscle cells. Neither GH nor BA treatment affected the activities of either of these enzymes and therefore we conclude that the observed changes in MyHC expression and metabolism were independent of changes in activity of the CaN/ calmodulin kinase pathway in this study. Obviously we cannot discount that effects of these agents on this pathway may occur before or after 6 days.

#### Metabolic regulatory factors

Transcriptional co-activators of the PGC-1 family, as well as the co-repressor RIP140, have previously been shown to influence the metabolic capacity of muscle fibres, with PGC-1 increasing oxidative metabolism and RIP140 decreasing it (Lin, 2009). Indeed, they appear to have opposite effects and actually counter each other in their transcriptional regulatory roles. In the current study, there was no effect of BA treatment on PGC-1*α* mRNA expression, but PGC-1*β* mRNA was significantly decreased by BA at both ages, coincident with the observed decrease in oxidative metabolism. This might suggest that PGC-1*β*, rather than PGC-1*α*, plays an important role in regulating the BA-induced changes in oxidative metabolism in sheep muscle. The lack of an effect on PGC-1*α* mRNA expression was unexpected as it is thought to mediate the effects of exercise on muscle, which involves *β*2-AR stimulation (Miura *et al*., 2007). Indeed, administration of the *β*2-adrenergic agonist, clenbuterol, has previously been shown to decrease PGC1*α* mRNA and protein expression, as well as oxidative metabolism, in rat muscles (Hoshino *et al*., 2011). However, administration of the endogenous *β*-adrenergic agonist, adrenaline, had no effect on the expression of PGC-1*α* mRNA in human muscle (Viguerie *et al*., 2004) and the authors suggested that the effects of adrenaline on PGC-1*α* expression may be species dependent. Our data would appear to agree with this suggestion.

RIP140 mRNA expression was increased by BA administration, which agrees with a recent study in rats (Hoshino *et al*., 2011). This increase, together with the decrease in PGC-1*β* mRNA, coincides with the decrease in oxidative metabolism, suggesting that these two factors are involved in the BA-induced metabolic response.

The PPAR nuclear receptors are targets of PGC-1 and RIP140 co-activation/co-repression and are thought to be major players in regulating lipid metabolism (Hock and Kralli, 2009). In the current study, BA increased PPAR*δ* mRNA expression, which may be involved in the increased energy expenditure observed previously in response to short-term BA treatment (MacRae *et al*., 1988). PPAR*α* expression was decreased in response to GH treatment, but what role/affect this has on muscle function or metabolism is still unclear. As the short-term administration of GH did not alter MyHC expression or any of the metabolic measurements in the LD, the lack of effects on the various signalling molecules is unsurprising. However, GH did have significant effects on PGC-1*β* and PPAR*α* mRNA expression, both of which are associated with metabolic changes (Hock and Kralli, 2009) and may therefore indicate that changes in muscle metabolism might be observed if GH were administered for longer. The altered expression of these metabolic regulatory genes in response to BA treatment gives additional support to the hypothesis that changes in MyHC isoform expression (and presumably fibre type transitions) are metabolically driven. The fact that changes in oxidative metabolism (ICDH activity) appear to precede any changes in protein levels of the MyHC isoforms, would support this. These key metabolic regulators may represent alternative signalling mechanisms for BA to the well-characterised signalling through *β*2-ARs and the cAMP activated second messenger pathway.

#### Six1 and Eya1

In recent years, a number of factors have been identified that regulate the slow oxidative muscle phenotype, particularly the stimulatory effects of the PGC-1 and PPAR pathways on oxidative metabolism and mitochondrial biogenesis and function. In comparison, relatively little is known about the factors that regulate the fast glycolytic phenotype. The transcription factor Six1 and cofactor Eya1, have been identified as initiating a shift in expression towards a faster muscle phenotype in mice (Grifone *et al*., 2004). However, our data would appear to contradict this as both factors were downregulated when the shift towards a faster, less oxidative phenotype was observed in LD in response to BA treatment. It has been reported that Six1 mRNA expression was downregulated in human skeletal muscle in response to lengthening contractions as early as 3 h after exercise (Kostek *et al*., 2007), whereas other genes associated with muscle hypertrophy (FHC1 and NEXN) were upregulated. Hence, Six1 may actually be a negative regulator of muscle hypertrophy that responds to BA treatment in sheep as well as to exercise in humans.

#### MRFs

The development and differentiation of muscle is dependent on the MRFs, MyoD, Myf-5, myogenin and MRF4. Muscle fibre number is thought to be fixed at birth in most large mammals and therefore postnatal muscle growth relates to fibre hypertrophy. This is thought to be dependent upon the differentiation and fusion of satellite cells to existing muscle fibres in order to increase the DNA content of fibres (Brameld *et al*., 1998). There are also suggestions that the MRFs may influence muscle fibre type composition, as MyoD mRNA expression is highest in fast glycolytic muscles of rats, whereas myogenin expression is highest in slow oxidative muscles (Hughes *et al*., 1993). BA administration significantly increased myogenin and decreased MyoD mRNA expression. As these changes coincided with the decrease in oxidative metabolism, the increase in MyHCIIX/IIB mRNA and induction of MyHCIIB mRNA, this would appear to contradict previous studies linking MyoD and myogenin mRNA expression with fibre type transitions in rats. However, as indicated above, the MRFs have other roles in skeletal muscle development, particularly satellite cell proliferation and fusion with muscle fibres during muscle regeneration and hypertrophy. One possible explanation for the described effects of BA in sheep muscle, is that BA increases satellite cell differentiation/fusion, which would be associated with increased myogenin and reduced MyoD expression (Brown *et al.*, 2012). In addition, the activities/functions of these transcription factors may be dependent upon their phosphorylation status rather than simply the relative mRNA or protein expression (Puri and Sartorelli, 2000).

## Conclusion

Over the 6-day time-course, BA had greater effects on muscle growth and phenotype than GH, especially in the older lambs. The BA-induced muscle growth was associated with changes in phenotype indicative of transitions towards a fast glycolytic fibre type in all muscles studied. The BA response included the induction of MyHCIIB mRNA expression, an isoform not normally expressed in sheep LD. These changes in MyHC expression corresponded with a decrease in oxidative metabolism (ICDH activity), which was likely induced by the combination of an increase in RIP140 and a decrease in PGC-1*β* mRNA expression. However, it remains unclear what (if any) factors are involved in driving towards the fast glycolytic fibre type, as expression of the previously suggested regulatory factors, Six1 and Eya1, was reduced by BA treatment. Our interpretation of the observations in this study are that changes in energy metabolism or substrate availability precede the changes in contractile proteins, but identification of the key factors involved in driving towards the fast glycolytic fibre type remains elusive.

## Supplementary material

To view supplementary material for this article, please visit http://dx.doi.org/10.1017/S175173111400233X
